# Aircraft measurement campaign on summer cloud microphysical properties over the Tibetan Plateau

**DOI:** 10.1038/s41598-019-41514-5

**Published:** 2019-03-20

**Authors:** Yi Chang, Xueliang Guo, Jie Tang, Guangxian Lu

**Affiliations:** 1 State Key Laboratory of Severe Weather (LASW), Chinese Academy of Meteorological Sciences, Beijing, 100081 China; 2 University of Chinese Academy of Sciences, Beijing, 100049 China; 3 Key Laboratory for Cloud Physics, China Meteorological Administration, Beijing, 100081 China; 4 Collaborative Innovation Center for Meteorological Disasters Forecast, Early Warning and Assessment, Nanjing University of Information Science and Technology, Nanjing, 210044 China

## Abstract

We reported the first aircraft campaign on summer cloud microphysical properties conducted in July of 2014 over the Tibetan Plateau during the third Tibetan Plateau Atmospheric Sciences Experiment (TIPEX-III), and demonstrated that the summer clouds over the Tibetan Plateau were primarily characterized as mixed-phase cumulus clouds induced by strong solar radiation heating. Moreover, the characteristic number concentration of cloud droplets (2~50 μm in diameter) in developing cumuli was around 10 cm^−3^, which was about 1~2 orders of magnitudes lower than other continent and ocean regions, and that for large drops (>50 μm in diameter) was around 10^−3^ cm^−3^, which was also lower than other regions. The droplet spectrum distributions (DSDs) of cloud drops were much wider than other regions, indicating that the cumulus clouds over the plateau could form precipitation easier than that in other regions. Ice microphysics was characterized as very active glaciation and riming processes with high supercooled water content, which caused the formation of high concentration of graupel particles in clouds. The findings of this study suggest that these unique cloud microphysical properties formed by the high topography and clean environment of the Tibetan Plateau could induce higher precipitation efficiency when airflow passed over the plateau, so that the plateau could act as a regional “water tower”.

## Introduction

Located in the southwestern region of China, the Tibetan Plateau (TP) has always been called the ‘roof of the world’. Its mean altitude exceeds 4000 m ASL (Above Sea Level), with the magnitude about 1000 km in meridional and 2500 km in zonal, it covers almost one quarter of total land area of China. Due to its huge area and high altitude, the TP plays an important role in regional and global climatology and meteorology. As there are many big rivers originating from the TP, its essential role in water cycles leads another description—‘Asian water tower’^1^. The thermal and dynamic circulation caused by the TP interdicts the Hadley Circulation, allowing water vapor originated from tropical ocean to transport into subtropical and middle and high latitude areas^2^. The TP also acts as a transport platform of water vapor from low latitude and is important for the formation of Meiyu rains^3–7^. The special effect of dynamics and thermodynamics induced by the TP contributes to the Asian monsoon’s onset and maintenance, and has significant effect on the climatology not only in eastern Asia but also in global^2,8–13^.

Cloud is a key process in atmospheric hydrological cycle and plays an important role in atmospheric energy budget. However, we know very little about clouds over the TP due to lack of relevant observations. Some previous investigations of clouds over the TP mainly depended on satellite measurement, and focused on cloud climatology^6,14–16^. Due to the low temporal and spatial resolution as well as the capability of satellite measurement, the detailed cloud microphysical properties such as ice habit, size distribution and relevant formation processes are hardly retrieved and verified. Furthermore, the satellite observation may exist malfunction and produce large uncertainties due to the effect of unique terrain of the TP^17^.

The preliminary radar observations in the second Tibetan Plateau Experiment of atmospheric science (TIPEX-II) at Naqu station showed that convective clouds were mostly column cells and the “pop-corn-like” convective clouds were always detected from satellite images^14^. The TRMM Precipitation Radar data also showed that the precipitation clouds tended to have a tower mast shape over the TP^15^. Recent Doppler radar observations showed that the summer clouds and precipitation had an obvious diurnal variation associated with strong solar radiation heating over the TP^11,18,19^. The droplet spectrum distribution (DSD) varied with the type and strength of clouds and precipitation, and had a wider spectrum that might be related to the higher falling speed due to the low air density over the TP^20–23^.

The cloud microphysical process over the TP remains unknown, resulting high discrepancies in modeling results^24,25^. The cloud microphysical characteristics and precipitation formation process are also essential for understanding the water cycle, heat budget, and mass exchange between troposphere and stratosphere over the TP. However, as the direct method to investigate cloud microphysics, the aircraft measurement has not been employed in the interior region of the TP. So, we launched the first aircraft measurement campaign on clouds in July of 2014 during the third Tibetan Plateau Atmospheric Sciences Experiment (TIPEX-III). The data and results of aircraft measurement are important in achieving cloud microphysical features, understanding the precipitation formation processes, improving and validating the microphysical parameterization schemes in numerical models and remote sensing retrieval techniques over the TP.

## Results

**Figure 1:**
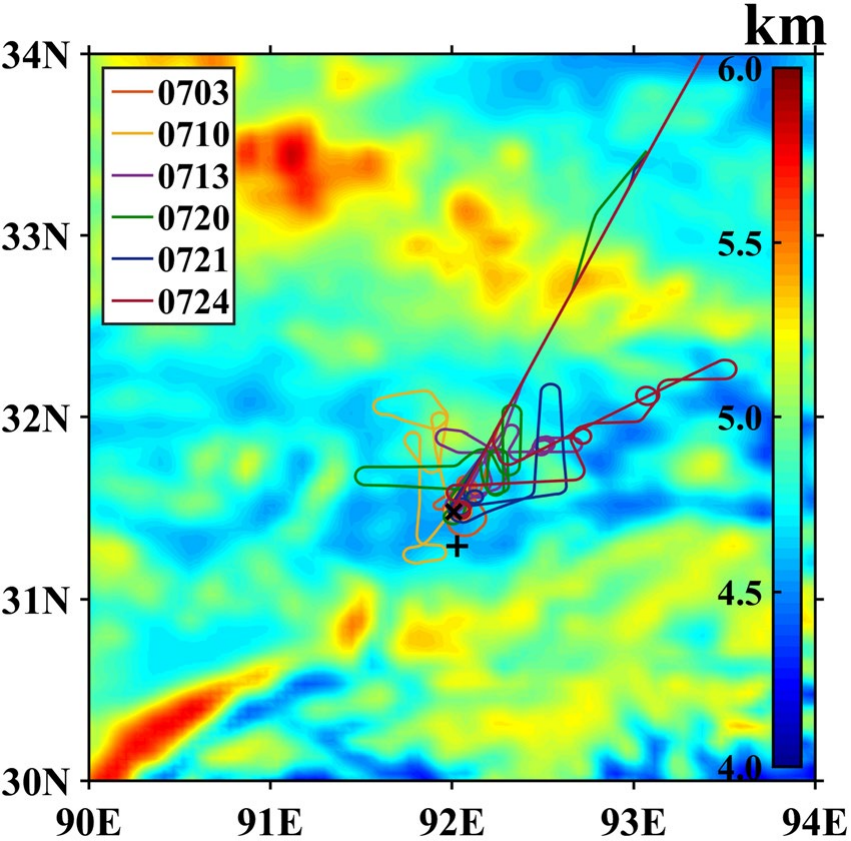
Terrain of observational region (filled contour), position of radars (black marker), and tracks of aircraft campaign (colored lines).

**Figure 2:**
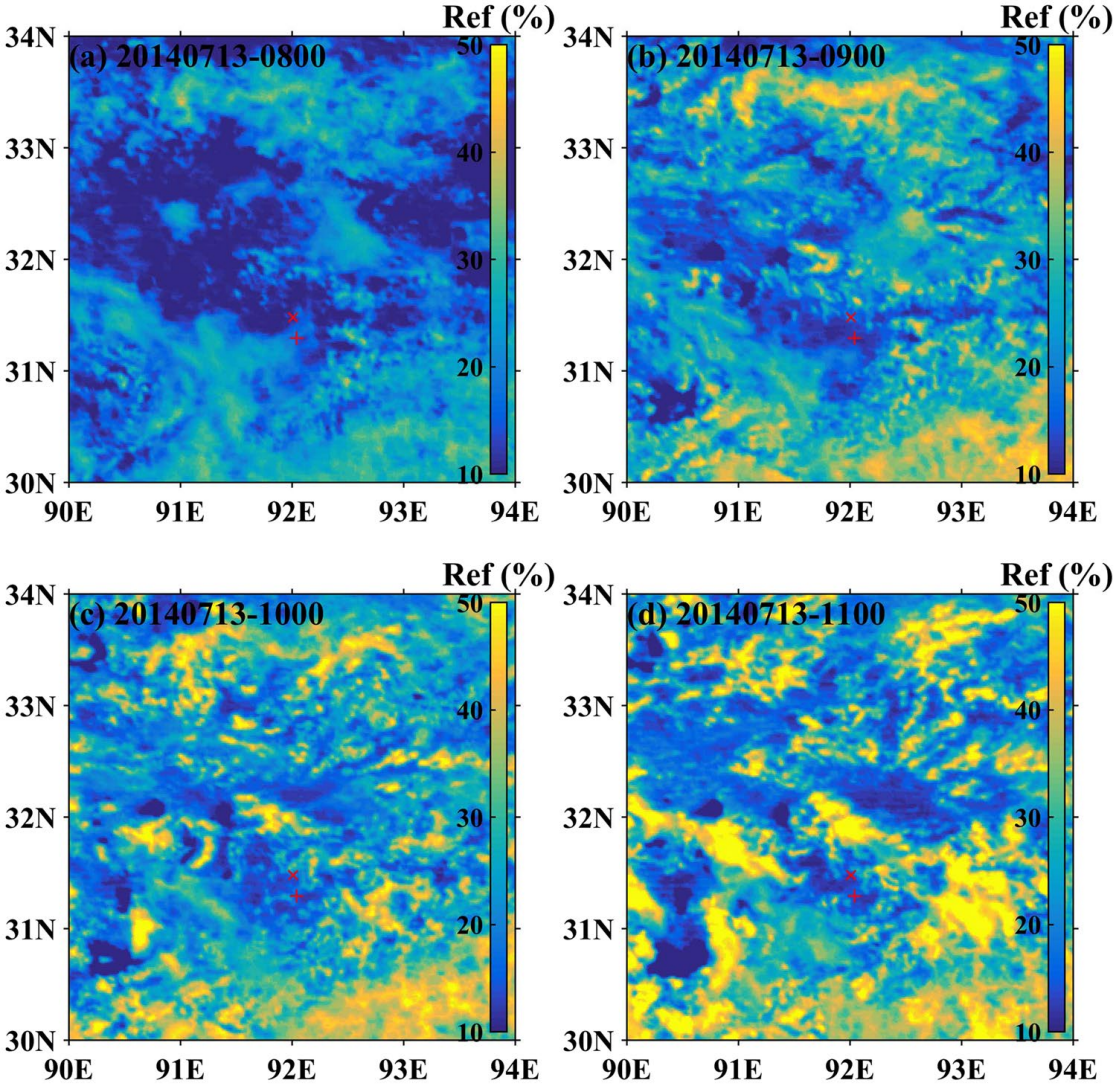
An example of cloud development and evolution over the TP in the forenoon, represented by the visible images of FY2E satellite (Ref is the abbreviation for reflectivity) of 08:00~11:00 LST (**a**–**d**) on 13 July, 2014.

The aircraft measurement campaign was conducted in July of 2014 by using aircraft King Air 350ER with probes of cloud microphysics. The flight tracks of total six cases and the location of Ka-radar, C-radar and Doppler radar are shown in Fig. 1. During the aircraft campaign, the clouds were singular convective cells formed by strong local heating in the late morning over the TP^11^, and then merged and developed as mesoscale cloud systems in the afternoon due to the influence of synoptic systems of shear lines and vortexes^5,20,26,27^. These processes could be clearly seen on the visible images of FY2E satellite in Fig. 2. The aircraft measurements were mainly conducted in the developing cumuli clouds. The clouds aircraft penetrated during the campaign were as follows:

0703: Newly born convective bubbles at ≈5740 m (−1.1 °C).

0710: Convective clouds with little or no precipitation at ≈6300 m (−2.5 °C) and ≈6900 m (−6.3 °C).

0713: Singular deep convective cells with ice and precipitation at ≈6300 m (−2.4 °C), ≈6600 m (−4.3 °C), and ≈7850 m (−12.9 °C).

0720: Residual clouds of previous deep convections at ≈8900 m (−16.9 °C) and singular deep convective cells with ice and precipitation at ≈6950 m (−5.5 °C).

0721: Weak residual mixed-phase cumulus clouds of previous deep convections at ≈8900 m (−17.2 °C) and continuous multi-layer mixed-phase clouds at ≈6300 m (−2.5 °C) and ≈6840 m (−5.7 °C).

0724: Continuous shallow convective clouds with precipitation below and a continuous boundary-layer clouds from afternoon at 23 July, the flight penetrated clouds at ≈6300 m (−4.5 °C), ≈6840 m (−7.1 °C) and ≈7380 m (−11.0 °C).

**Figure 3:**
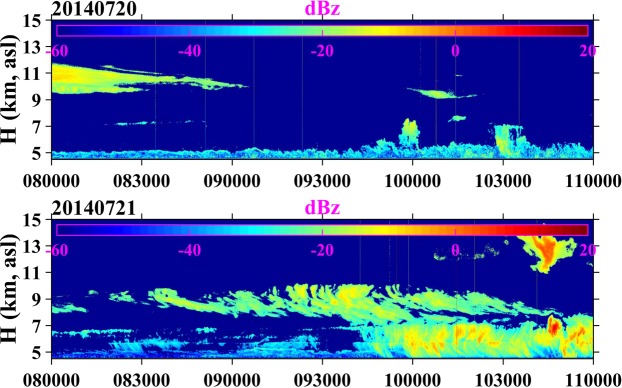
Time evolution of clouds observed by C-radar from 08:00 to 11:00 LST on July 20 (top) and 21 (bottom), 2014.

We used two vertically pointing radars to observe the vertical structure of clouds over the Naqu station during the campaign. Figure 3 shows two examples (20 and 21, July) of cloud vertical structure obtained by C-radar, and the two examples contained all cloud types during the campaign. On 20 July, the residual convective clouds were covered above 9 km, and the singular convective cells started to develop around 10:00 LST (Local Standard Time). On 21 July, the clouds passing over Naqu station showed a two-layer structure; before 10:00 LST, the cloud near Naqu station was weak cumulus distributed from 7 km to 10 km, which should be formed by synoptic system. At around 9:45 LST, the weak convective cells were developed from low levels associated with solar radiation heating.

During the aircraft campaign, the observed maximum cloud droplet concentration was 1.1 × 10^2^ cm^−3^ at 6300 m (**−**2.5 °C) in the convective cell on 10 July, and the cloud droplet concentration was in order of magnitude of 10^1^ cm^−3^. Large particles were measured by 2D-S (Two-Dimensional Stereo Optic Array Spectrometer) and HVPS (High Volume Precipitation Spectrometer) probes, since the HVPS probe was stable and reliable during the observation period, we used the HVPS probe as the measurement of large particle concentration. The observed maximum large particle concentration was 2.9 × 10^−2^ cm^−3^ at 7850 m (**−**12.9 °C) on 13 July, and had an order of magnitude of large particle concentration was 10^−3^~10^−2^ cm^−3^. By analyzing the 3V-CPI (Three-View Cloud Particle Imager) images, we found that the drizzles were corresponding to an order of magnitude of 10^−3^ cm^−3^ and the ice particles were corresponding to the value of 10^−2^ cm^−3^, however, owing to insufficient data sets, this correspondence could not be fully certified. Vertical velocities were mainly ranging from 1 m/s to 4 m/s, and some weak cumulus clouds had the vertical velocity of lower than 1 m/s. We also examined the CAPE (Convective Available Potential Energy) of Naqu station during the aircraft campaign, and found that the CAPE values at 06:00 LST of all six cases were quite small ranging from 0.00 J/kg to 129.35 J/kg. The low values of vertical velocities and CAPE indicated that the clouds over the TP during the aircraft measurement campaign were weak or in the stage of developing cumuli.

**Figure 4:**
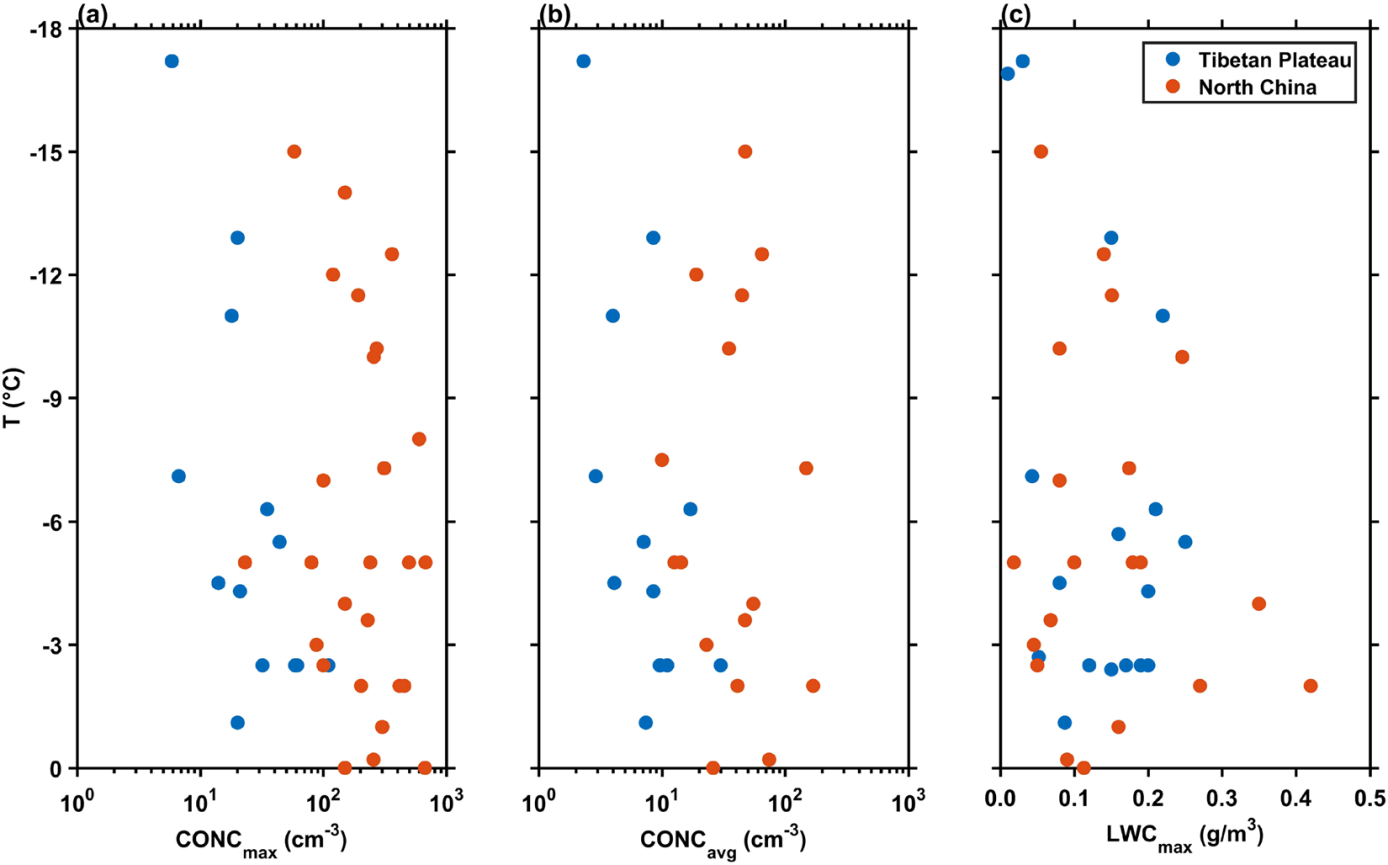
Comparisons of (**a**) maximum cloud drop concentration, (**b**) average cloud drop concentration, and (**c**) liquid water content in clouds between the Tibetan Plateau and Northern China.

The comparisons of vertical distributions of microphysical parameters observed over the TP and northern China^28–34^ are shown in Fig. 4. In Fig. 4a,b, we can clearly see that the maximum and average cloud droplet concentrations over the TP were 1~2 orders of magnitude smaller than that over the northern China at same temperature levels. However, the liquid water content (LWC) over the TP had the same order of magnitude with that over the northern China. The maximum LWC of 0.25 g/m^3^ at 6953 m (−5.5 °C) on 20 July was relatively larger than that over the northern China at same temperature. Table 1 lists the comparison of cloud droplet concentration between the TIPEX-III and other campaigns^35–40^. It is obvious that cloud droplet concentrations in clouds over continents were generally larger than that over sea. However, the cloud droplet concentration over the TP was even much smaller than that over sea.

**Table 1:** Comparison of droplet concentration in warm clouds between the TIPEX-III and other campaigns.

Project	Season	Air mass type	Concentration (cm^−3^)
TIPEX-III	Summer	Continental	9 ± 10
FIRE	Summer	Maritime	39 ± 24
ASTEX	Summer	Continental	188 ± 114
		Maritime	91 ± 60
SOCEX1	Winter	Maritime	25 ± 23
SOCEX2	Summer	Maritime	69 ± 56
ACE1	Summer	Maritime	63 ± 63
SCMS	Summer	Continental	241 ± 202
		Maritime	122 ± 117

**Figure 5:**
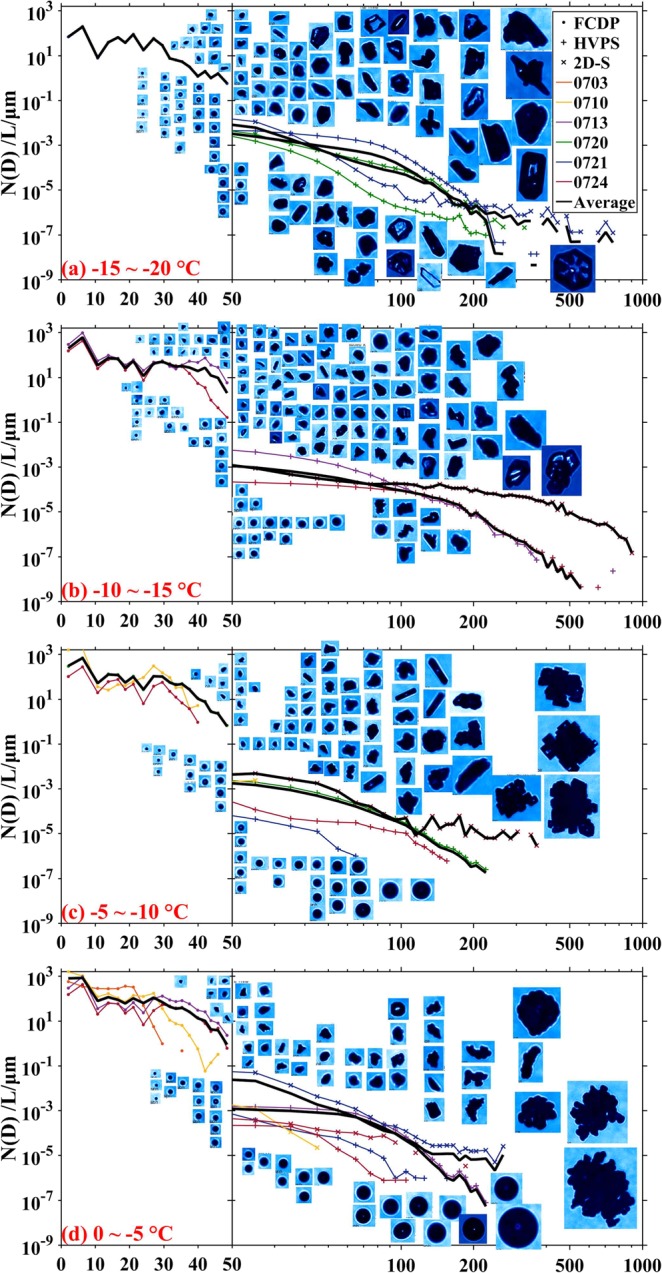
Particle size distributions and sampled particle images at layers of (**a**) −15~−20 °C, (**b**) **−**10~**−**15 °C, (**c**) **−**5~**−**10 °C and (**d**) 0~**−**5 °C.

Besides the much lower concentrations of cloud droplets over the TP than that over the other regions, the particle size distributions over the TP also showed some quite different features. Figure 5 shows the variations of particles size distributions and sampled particles images at different temperatures. We can see clearly from the figure that DSDs over the TP had similar spectrum shape at different altitudes (temperatures), all of DSDs had the first peak at 6.3 μm (mean value of the bin between 3.2 μm and 9.4 μm) and with multimodal shape in the large diameter range (20~50 μm). Compared with other cumulus researches^38,41–43^, the spectra were much wider and with more peaks over the TP than those over the other regions.

The cloud drops of less than 50μm were observed by 3V-CPI at all altitudes (temperatures), even at the high level of nearly 9000 m (**−**17 °C) (Fig. 5a). Many large cloud drops were observed in the lower altitudes (0~−10 °C). Raindrops (diameters larger than 200 μm) were also observed in 0~−5 °C. This phenomenon indicates that there were many supercooled cloud drops in the clouds over the TP.

Ice particle distributions had two apparent features, one is that at high levels between −10~**−**20 °C the freezing and riming processes were active in clouds, which provided a favorable condition for the formation of graupel particles. Another is that at low levels between 0~−10 °C both riming and aggregation processes were active in clouds. The DSDs of larger particles over the TP showed an exponential shape. A key feature of ice particles over the TP was the high content of graupel particles which account for a large portion of ice particles in all temperatures. In Fig. 5, many ice particles were opaque and wet, and they seemed to have absorbed cloud drops, indicating an active riming process in clouds.

Regular shaped particles were not frequently observed in clouds over the TP, indicating that the formation of prime ice particles mainly depended on the freezing process of cloud drops. Bullet crystals were found at temperature of **−**17 °C accompanied with few rosette polymers (Fig. 5a). Plate-like hexagonal crystals emerged at temperature lower than **−**10 °C. Column crystals were detected at −5~−10 °C, which was consistent with previous investigations^44,45^. In higher altitudes, small needle crystals were also obtained. Irregular solid ice crystals mostly occurred at altitude with temperature lower than **−**15 °C, this is mainly because that in higher altitudes the liquid water content was relatively low, and the riming process decreased. Glaciation process was frequent in clouds over the TP, especially for ice crystals smaller than 50 μm.

## Discussions

Our results show that cloud droplets over the TP had a much lower concentration and wider spectrum than other continent and ocean regions, indicating that the clouds make the rain much easier over the TP than that in other regions. This interesting phenomenon indicated that the air over the TP is very clean and thus resulting in fewer cloud condensation nuclei (CCN) and larger cloud droplets. Moreover, the ice processes relevant to graupel formation and riming process were very active in the clouds over the TP. Due to the high altitude and low temperature, the observational cloud bases over the TP were near to the ground and the temperatures of all sampled clouds were lower than 0 °C for six cases, and the sampled clouds over the TP were mostly in a mixed-phase state. Since former researchers rarely studied particle habits and phase conversion processes over the TP due to lack of direct observational data, our study firstly demonstrates that graupel formation and riming processes were very active in clouds over the TP.

This work provided the first evidence for cloud microphysical properties over the TP, which was essential for improving the parameterization of cloud microphysics in numerical models and validating remote sensing data in this region. However, due to the limitation of aircraft capability in taking off, landing and loading over the TP, the flight time of aircraft observations in this campaign were relatively short and the observations could not continue in the whole lifetime of clouds. Thus, the further development of clouds in the afternoon and nighttime could not be continuously probed. It is necessary to fully investigate the properties of cloud microphysics and precipitation formation in the whole lifetime of clouds in the future aircraft measurement campaign over the TP.

## Methods

We conducted an intensive measurement campaign combined with ground-based and aircraft instruments to investigate the physical properties of clouds and precipitation from 1 July to 31 August 2014 at Naqu region in the central TP during TIPEX-III. Aircraft campaign was held in July, 2014, the six valuable cases were obtained in total of 12 cases from 1 to 24 July. All cases were observed between 7:00 and 11:00 LST.

To diagnose the synoptic systems during aircraft measurement campaign, the ECMWF ERA-Interim reanalysis data with 0.125 ° × 0.125 ° spatial resolution and 6-hour temporal resolution was employed. The geostationary satellite of FY2E full-size data with spatial resolution of 1 km and 5 km for visible and infrared channels, and temporal resolution of 0.5 or 1 hour were used. The Ka-band millimeter wave cloud radar data with spatial resolution of 30 m and temporal resolution of 8.797 s, the C-band continuous wave radar data with spatial resolution of 30 m and temporal resolution of 3 s, and CMA operational Doppler radar data were also employed. The Ka-band and C-band radars were vertically pointing and more detailed technical parameters can be found in published papers^20,46,47^. The temperature profiles were determined by intensive air sounding data.

The aircraft employed during observation campaign was King Air 350ER from the Beijing Weather Modification Office (BJWMO) with airborne probes from SPEC (Stratton Park Engineering Company: http://www.specinc.com). The probes used in this study include the Aircraft Integrated Meteorological Measurement System (AIMMS-20); the Hotwire Liquid/Total Water Content (LWC/TWC) measurement Nevzorov; the Two-Dimensional Stereo Optic Array Spectrometer (2D-S); the Fast Cloud Droplet Probe (FCDP); the High Volume Precipitation Spectrometer (HVPS), and the Three-View Cloud Particle Imager (3V-CPI). The detailed technical parameters can be found in Table 2.

**Table 2:** Technical parameters of probes on King Air 350ER.

Probe	Variable	Range	Resolution
AIMMS-20	Temperature	−54~71 °C	0.5 °C
	Altitude (ASL)	0~13.7 km	18.3m
	Position (GPS)	N/A	10 m
	Air speed	0~220 m/s	1 m/s
Nevzorov	LWC/TWC	0.005~3 g/m^3^	0.005 g/m^3^
2D-S	Cloud and precipitation particles	10~3000 μm	10 μm
FCDP		1.5~50 μm	3 μm
HVPS		150~19200 μm	10 μm
3V-CPI		7~3000 μm	2.3 μm

## Data Availability

All data is available on-line and free of charge. The ECMWF ERA-Interim reanalysis data is available at the European Centre for Medium-Range Weather Forecasts. The CMA operational Doppler radar data and the datasets of TIPEX-III can be found at China National Meteorological Data Service Center (CMDC). The observational data of TIPEX-III is available at http://data.cma.cn/tipex.
